# Synergy of Microfluidics and Ultrasound: Process Intensification Challenges and Opportunities

**DOI:** 10.1007/s41061-016-0070-y

**Published:** 2016-09-21

**Authors:** David Fernandez Rivas, Simon Kuhn

**Affiliations:** 1 Mesoscale Chemical Systems, MESA+ Institute for Nanotechnology, Carre 1.339, 7500 AE Enschede, The Netherlands; 2 Department of Chemical Engineering, KU Leuven, Celestijnenlaan 200F, 3001 Leuven, Belgium

**Keywords:** Microfluidics, Ultrasound, Sonochemistry, Process intensification, Chemical engineering, Solids handling

## Abstract

A compact snapshot of the current convergence of novel developments relevant to chemical engineering is given. Process intensification concepts are analysed through the lens of microfluidics and sonochemistry. Economical drivers and their influence on scientific activities are mentioned, including innovation opportunities towards deployment into society. We focus on the control of cavitation as a means to improve the energy efficiency of sonochemical reactors, as well as in the solids handling with ultrasound; both are considered the most difficult hurdles for its adoption in a practical and industrial sense. Particular examples for microfluidic clogging prevention, numbering-up and scaling-up strategies are given. To conclude, an outlook of possible new directions of this active and promising combination of technologies is hinted.

## Introduction and Definitions

### The Basics of Ultrasound

**Fig. 1:**
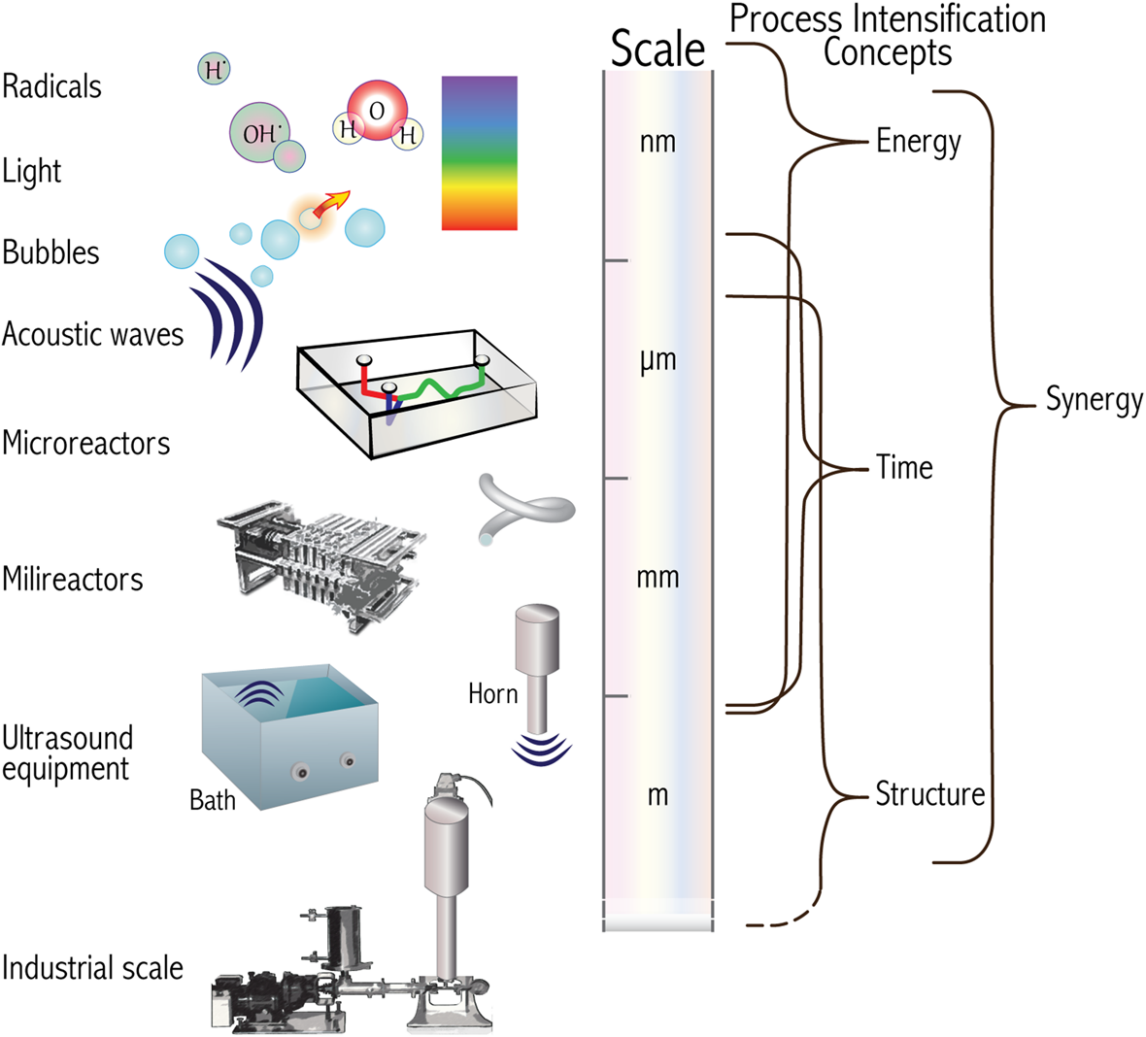
Diagram with terms and concepts described in this chapter. The relative sizes of the items described on the *left* increase from top to bottom. On the *right *we connect the different sizes where the process intensification concepts have the greatest influence

We present in Fig. 1 a comprehensive diagram with terms and concepts that will be described and expanded in the text. After reading this work, it will be clearer to the reader how synergy, understood in the framework of process intensification (PI), has the largest relevance when analysing the combination of microfluidics and ultrasound applications.

Ultrasound effects on matter are widely known in academic and industry circles. There are several practical applications that have been extensively covered in the literature, ranging from mechanical and chemical processing, medicine, food industry, to cleaning. Use of ultrasound can be either continuous or pulsed and across a broad range of frequencies (20 kHz up to 1 MHz) and acoustic pressures depending on the application  [1–6]. Frequencies higher than 1 MHz are known to be used, especially when talking about particle handling and acoustic streaming [7, 8]. Sonochemistry describes the chemical effects of ultrasound on molecular transformations. Different from other energy sources to drive reactions, the chemical effect of ultrasound in liquids is not linked to a direct interaction with the molecules [9]; instead, the energy contained in the sound is channeled into acoustic cavitation, i.e. the formation, growth, and subsequent collapse of bubbles in a liquid [10]. These collapse events lead to temperature and pressure hot spots inside the bubble (high temperatures >5000 K and pressures >1000 atm), which in turn activate chemical transformations without altering much the liquid medium [9, 11]. Actually, although the high temperatures and pressures are inside the bubble, most of the relevant chemistry happens in the liquid phase. The importance of bubble collapse is such that it appears that chemical reactions as a result of cavitation might have played a role in producing complex organic molecules in prebiotic times [12]. We can fairly say that ultrasonication, or the exposure of matter to ultrasound, has reached a state of maturity.

Despite the wide use of cavitation, the borders between the physical and chemical phenomena remain blurred, which contributes to an extended use of sonochemistry in a *black-box* manner. The reason behind this is that cavitation effects such as of liquid jets, streaming, chemical radical molecules production, plasma formation with light emission, and shockwaves, are intertwined in complex dependencies that are hard to resolve in space and time [13]. Often, users of sonochemistry and outsiders tend to confuse the definition of a bubble (gas in a liquid, or gas in gas as in soap bubbles) and cannot correlate a given effect with a specific phenomenon. This situation has not prevented sonochemistry from being widely exploited in several useful applications, yet we think it could be done more efficiently.

### The Basics of Microreactors

Using microstructured devices in chemical engineering provides several advantages over conventional, and mostly batch, reaction systems. Because of the decrease in characteristic length scale, an increased surface-to-volume ratio $(\mathrm {m}^{2}/\mathrm {m}^{3})$ is obtained, with benefits such as enhanced heat and mass transfer coefficients, as well as improved energy conversion efficiencies [14–19]. In addition, the typically small volumes allow a safer handling of hazardous materials and reduced risk when performing high-parameter reactions (pressure and temperature). Early studies showed the potential of using microreactors for chemical synthesis in small-scale flows [14, 15]. Research over the past decade focused on developing complex microchemical systems to enable multi-step processes, especially focusing on transformations involving multiphase flows. A prime example of such a multi-step microchemical synthesis is the continuous flow, multi-step Heck synthesis performed by integrating microreactors, liquid–liquid extraction, and microfluidic distillation [20]. These early studies highlight the potential and the usability of microchemical devices, especially for rapid experimentation and shortening product development cycles.

In order to define the size range of microchannels or capillaries it is not sufficient to consider only their characteristic dimension, e.g.  in terms of their hydrodynamic diameter $D_\mathrm{H}$. Also, the wall roughness of microfluidic devices $(D_\mathrm{H}\sim \mathcal {O}( 10^{-6} \,\mathrm {m}))$ cannot be neglected at this scale, and it is associated with the tolerances of microfabrication techniques [21]. The roughness and hydrophobic properties of a surface have a strong influence in the existence of small gas pockets that can eventually serve as nucleation of bubbles, as will be discussed further in this text. The existence of surface nanobubbles is related to the pinning of the three-phase contact line at chemical or geometric surface heterogeneities, and such bubbles can form when the liquid comes into contact with the surface, but also from gas supersaturation during the immersion process [22].

On the other hand, concentration, temperature, and other gradients are reduced significantly when compared to equivalent conventional devices. Singular behaviour of matter at the microscale is rather attributed to the application of models derived for large-scale geometries, describing pressure drop and transport processes (e.g. wall heat transfer) that deviate considerably when applied to microchannels.

An important observation of chemical processes in microchannels is that the two-phase flow regime is independent of the channel orientation, i.e. the phase distribution will be the same in horizontal and vertical channel alignment. These differences between macro- and microchannels arise from the relative importance of gravity and interfacial forces: interfacial effects dominate phenomena at the microscale. Consequently, the definition of microchannels does not just depend on their physical size, but also on the properties of the considered fluid system. One proposed criterion is to calculate the Laplace length scale $\lambda$ [23], which uses the ratio of interfacial and gravitational forces to quantify a cut-off dimension below which the effect of gravity can be neglected

1 $$\begin{aligned} \lambda =\sqrt{\frac{\sigma }{g( \rho _L-\rho _\mathrm{G}) }} \end{aligned}$$

where $\sigma$ denotes the interfacial tension, *g* is the acceleration due to gravity, and $\rho _L$ and $\rho _\mathrm{G}$ are the densities of the liquid and gas (or second immiscible liquid) phase, respectively. If the hydrodynamic diameter of the flow channel is smaller than this Laplace length scale ($D_\mathrm{H} < \lambda$) it can be considered a microchannel, and this dependence on the fluid properties results in a rather wide size range.

**Table 1:** Fluid properties and associated Laplace length scale for selected two-phase flow systems [24, 25]

Property	Water	Nitrogen	1-Butanol	Toluene	DMSO
Density $\rho$ (kg m$^{-3}$)	998	1.25	810	867	1100
Interfacial tension $\sigma$ (N m$^{-1}$)		0.072	0.002	0.037	0.057
Laplace length scale $\lambda$ (mm)		2.71	0.98	5.33	7.65

Values for the interfacial tension and Laplace length scale assume water as the primary phase

Table 1 shows selected (and commonly encountered classes) of two-phase flow systems and their corresponding values of the Laplace length scale. Gas-liquid systems are characterized by large density differences, but also large interfacial tension, which then results in Laplace constants of $\lambda \sim 3 \,\mathrm {mm}$. In liquid–liquid systems the determining parameter is interfacial tension, as the density difference between two liquid phases is negligible. Table 1 quantifies one example each for low (1-butanol), intermediate (toluene), and large (DMSO) interfacial tension, and the accompanying wide size range below which gravitational effects can be neglected.

**Fig. 2:**
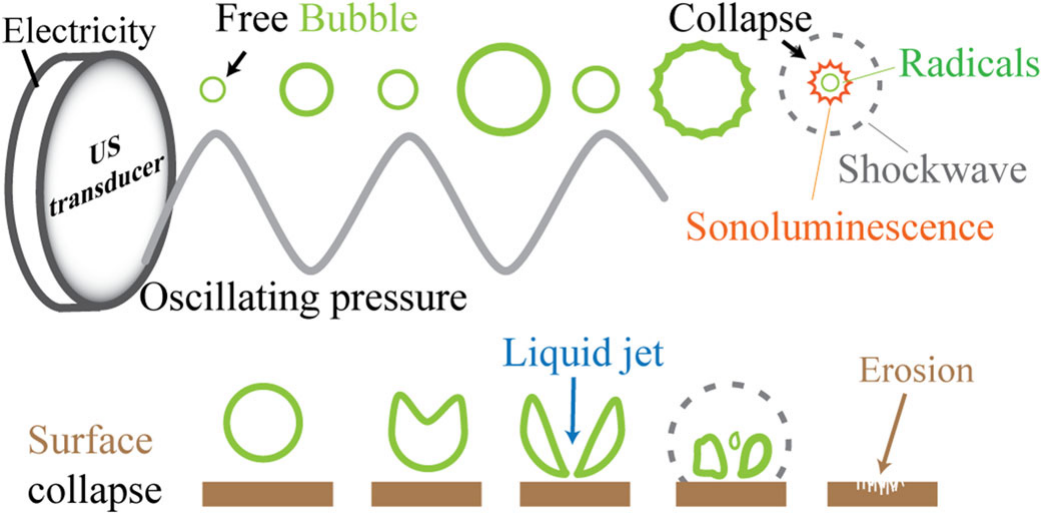
Schematic representation of a free bubble and a bubble close to a wall, oscillating due to pressure variations imposed by an ultrasound transducer. At the moment of collapse, radicals, shockwaves and light emission (sonoluminescence) can be produced as detailed in the text. When near a surface or another bubble, jetting can also occur. Repeated collapse events against a surface can lead to erosion of surfaces

It is known that acoustic cavitation relying on nuclei does not occur easily in microfluidics because the static pressure inside the microchannels is high, which tends to promote the fast dissolution of gas bubbles [26]. For a cavity or bubble to be formed in a liquid, the energy required theoretically is extremely high. For a small bubble forming in water with a radius ~10^−10^ m, the minimum negative pressure (also referred to as Blake threshold pressure) required to overcome water molecule cohesion forces is ca. 1400 atm [27]. The pressure inside a gas bubble $p_\mathrm{g}\left( t \right)$ is a result of the sum of the ambient pressure $P_{0}$ and the Laplace pressure (not considering the vapor pressure)

2 $$\begin{aligned} p_\mathrm{g}\left( t \right) =P_{0}+\frac{2\sigma }{R\left( t \right) } \end{aligned}$$

where $\sigma$ is the surface tension and $R\left( t \right)$ is the bubble radius. When the first term in the right hand side of Eq. 2 dominates, bubbles can be considered “large”, meaning that the gas pressure inside the bubble is dominated by the ambient pressure; as a reference, $R\gg 2\sigma /P_{0}$, corresponds to a radius $R \approx 1.4 ~\upmu \mathrm{m}$, for air in water at ambient pressure. For small bubbles the Laplace pressure dominates. During the expansion phase (lower pressure) of a sound wave, bubbles can be formed (see Fig. 2).

In practice, values much smaller than the Blake threshold pressure (1–3 atm) are sufficient to create bubbles [28]. This is due to the presence of small particles or defects in the container holding the liquid, that can entrap gas nuclei, which serve as a weakening spot for molecular cohesion. Once a bubble is formed and reaches a resonance size depending on the ultrasonic frequency, it can suddenly grow to a larger size, become unstable, and violently collapse, hence the term transient cavitation. Alternatively the bubble may oscillate during several cycles at its resonance size, meanwhile generating large local streaming; this is known as stable cavitation. Acoustically driven capillary waves can also induce cavitation in microfluidics. Capillary waves travel on the surface of a liquid while restoring forces are provided by the interfacial tension when the wavelengths are short enough for gravity to be neglected. There are comprehensive works covering several aspects of the types of bubbles, and cavitation, for which we refer to the literature [22, 26, 27, 29].

### Synergistic Effects When Combining Ultrasound with Microreactors

The use of the term synergy is not always correct in specialised literature. Most definitions or examples fail to illustrate how two or more techniques are able to produce a combined effect greater than the sum of their separate effects, particularly in sonochemistry [30–32]. The concept of process intensification is highly linked to modern chemical engineering as it aims for a more sustainable and efficient way to manufacture chemical products [33]. This is achieved by the introduction of innovative principles in both process and equipment design, which will then lead to significant improvement in process efficiency and product quality, that in turn further reduces waste streams.

**Fig. 3:**
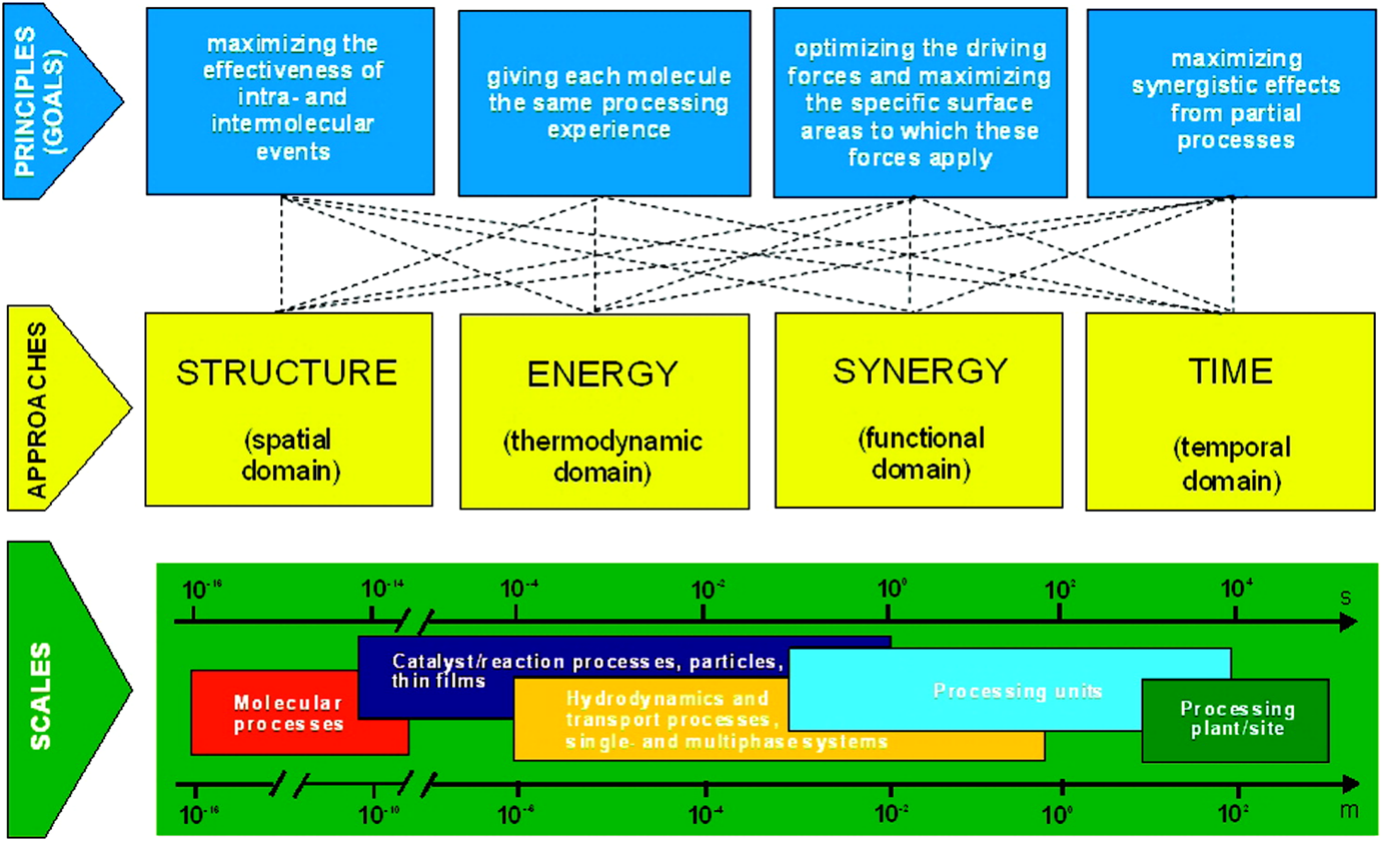
Fundamental view on process intensification showing the connection between the involved scales, approaches and goals [33]. The approaches to successfully intensify a process can be categorized in four domains: spatial, thermodynamic, functional, and temporal. These approaches need to be applied on all the relevant time and length scales depicted in the green bar. These scales range from the molecular level to the size of a chemical plant. Reprinted with permission from [33]. Copyright 2009 American Chemical Society

Figure 3 depicts the main goals of PI together with the associated length scales:

1. Maximize the effectiveness of intra- and intermolecular events,
2. Give each molecule the same processing experience,
3. Optimize the driving forces at every scale and maximize the specific surface area, and
4. Maximize the synergistic effects from partial processes.

Given its inherent properties, microfluidic systems can address all these goals, and in parallel chemical engineers have assimilated the fact that “larger” equipment is not always necessary to reach an economy of scale. In the past three decades several academicians and companies have adopted a “scaling-down” strategy of processes [34–36]. In addition to the benefits mentioned earlier in Sect. 1.2, microfluidic systems represent a well-defined reaction platform for a wide array of chemical manipulations and synthesis. Among other properties, the residence time of species and the reactor temperature can be precisely controlled [37, 38]. This also allows rapid experimentation in terms of high-throughput screening (e.g. reaction conditions, catalysts) [39–41] and automated optimization [42–44]. In addition, microfluidic systems open novel process windows [45], as they allow safe synthesis in harsh conditions [46] and increase the achievable reaction space by e.g. performing synthesis in supercritical solvents [47–49]. All these studies highlight the potential of using micro-reactors for chemical synthesis in small-scale flows, and consequently more complex microchemical systems to enable multi-step processes have been developed [20, 50].

Owing to the small length scales and associated small fluid penetration depths, microfluidic devices also allow the integration of various external energy sources, and thus open up novel routes for PI. Selected applications include photo- and electrochemistry [51, 52], and acoustic microfluidics [53]. While acoustic microfluidics is mostly related to the manipulation of particles via the acoustophoretic force [54], here we focus on the synergistic effects of microfluidics and ultrasound. Since quantifying the separate effects of using microfluidics with ultrasound and comparing the results with their combined effect is required to demonstrate a synergy, we perform a mind experiment. Take a microfluidic reactor that can operate for 1 h until it clogs. The application of ultrasound (even below the pressure amplitudes that yield cavitation) will have improved mixing, heat, and mass transfer, but no direct effect whatsoever in the reaction progress (radicals, light, etc). Most likely, the clogging of the microreactor will be reduced and the reactor will be able to operate for a longer time. This has a clear synergy result, in terms of mechanical effects, since the combination of both techniques will lead to the desired products and longer operation time.

Progress in the field of continuous manufacturing has also been well received by industry. In a collaborative paper co-authored by several major pharmaceutical and fine chemistry companies (GSK, DSM, Boehringer Ingelheim, Pfizer, AstraZeneca, Eli Lilly, Johnson & Johnson, Merck) the need for further research efforts in process intensification was already identified recently [55]. This also includes the demand for novel concepts for continuous reaction systems to enable fine chemicals manufacture.

Despite the many advantages of microchemical systems and their successful applications in chemical engineering research and pioneering companies, one major practical drawback greatly limiting their use in commercial environments and larger scales, is their susceptibility to channel clogging for flows containing particulate matter. This is especially problematic when solid particles are formed as a by-product of a particular chemical reaction, as the amount of solid matter gradually increases along the axial extent of the microchannel. Integrating microfluidic reactors with ultrasonic actuators has been proven successful to mitigate clogging and to ensure long-term operation [56–58]. We expect that more attention to the potential of combining microfluidics and ultrasound will be given in the coming years, and more studies able to highlight the “real” synergy will see the light. Other examples will also be given in Sect. 3.

## Economical Drivers and Scientific Impact

Fundamental transformations have taken place in the ways that society and the economy influence each other over the last decade. The current growth model has been exhausted as we observe a slowdown in the global economy. A possible alternative to the ways we produce and exploit the natural resources in a sustainable way can be supported by technological changes or innovations. Ecological aspects, such as the impact on the environment and other phenomena such as global warming and the realization that natural resources are not unlimited, has triggered a more conscious approach on how we can continue exploiting natural resources in a sustainable, safer, and responsible way. Ultrasound and microfluidics separately have contributed to significant advances in this respect [30, 59, 60]. Scientific innovation is being accelerated both in globalised markets and our more interconnected world, at a pace not reported before. Some experts credit the Fourth Industrial Revolution for which enabling technologies such as alternative fuels, 3-D printing, and nanotechnology, have already been linked to microfluidics and ultrasound [61–68]. On the other hand, with the current power of social media, some scientists team up with designers, marketing and other professionals across disciplines, in such a way that it is possible to address better the existing needs of society. This can result in a market-pull approach where new ideas and products are tailored to specific costumers, and in turn will accelerate deployment into society.

Currently, the ultrasonics and microfluidics communities are induced to innovate and revisit existing knowledge toolboxes in order to ensue a technology-push with the hope to commercialise new inventions. Since 1996, the number of research publications and patents having as keywords “microfluidics”, “chemistry”, and “ultrasound” indexed in Scopus is 1908 and 1262 respectively. The number of articles in 2015 was 357, and the number of patents was 128, with an estimated 10 % increase per year in both categories over the last 5 years. Microfluidics as a commercial activity is in its hype phase, where established companies and new spin-offs amount to an estimated 670 according to industrial observers [69]. An interesting trend is seen towards integration of platforms such as plug-and-play components and standardisation. Further, several universities are active in the creation of spin-offs that can eventually be bought by larger companies (e.g. pharmaceutical and electronic) depending on their potential to produce components, and the services they can provide.

We refer the reader to some references and interesting developments around microreactor technology given by Sigma-Aldrich’s product manager [70], including the possibility to buy generic “all-in-one solution” microreactors, which are labeled as “the chemist's round bottomed flask of the twenty-first century”. Several companies provide commercial micro- and milli-reactor solutions, e.g. Chemtrix, Corning, ESK, Micronit and Velocys. Among the companies involved in the commercialization of ultrasound integrated reactors we are aware of, Hielsher commercialises an Ultrasonic Mini flow cell and a GDmini2 Ultrasonic Inline Micro-reactor; Prosonix has catered to larger scale reactors in its Prosonitron project for Aughinish Alumina (Glencore) [71].

Conversely, some larger transnational companies are shedding their research and development groups that sometimes become start-ups on its own. The spun-out researchers that assemble in new companies toughen the competition between academics and other institutes for subsidies or research funds. Shorter product-life cycles, together with continuously changing market trends and offer-demand balance are the new normal in most commercial activities [72]. This situation has had a tremendous impact worldwide on the chemical industry and research as a whole, twisting, or skewing governmental financing towards private investment in many countries. Since subsidies and funding scheme for research are drying out, the advent of social campaigns has become an alternative. Researchers are increasingly more active in social media and stream their research and products to meet non conventional demands, such as for cooking [73, 74]. Non-traditional ways to fund research such as for innovative ultrasonic equipment for cleaning applications have been funded through crowd-sourcing schemes [75].

Nevertheless, there are certain risks that need to be addressed and minimized. To begin with, the regulations and amount of information ruling the use of US equipment are scarce. Logically, until they are not clarified, it will be hard to expand the current industrial applications of ultrasound and sonochemistry towards the consumers market with revolutionary ultrasonic equipment [76].

## Overcoming Challenges and Opening New Opportunities

### Integrating Ultrasound with Microfluidics

Going “micro” with ultrasound is not straightforward, neither is interpreting the results of experiments or processes, yet its potential for different chemical uses has already been identified [77]. Microfluidics enable the manipulation of chemical reactions using very small amounts of fluid, and ultrasound offers a good “non-invasive” alternative for several processes. Additionally, the small quantities of reagents, solvents, and waste, a precise control of reaction conditions, as well as the integration of functionality for process intensification, have all been highlighted as greener, safer, and often faster protocols [30]. On the other hand, answering the perennial question on how to process or produce larger volumes of liquids with microfluidics, several numbering-up and scaling-up strategies, as well as manifolding have been explored with varied success [78–81].

**Fig. 4:**
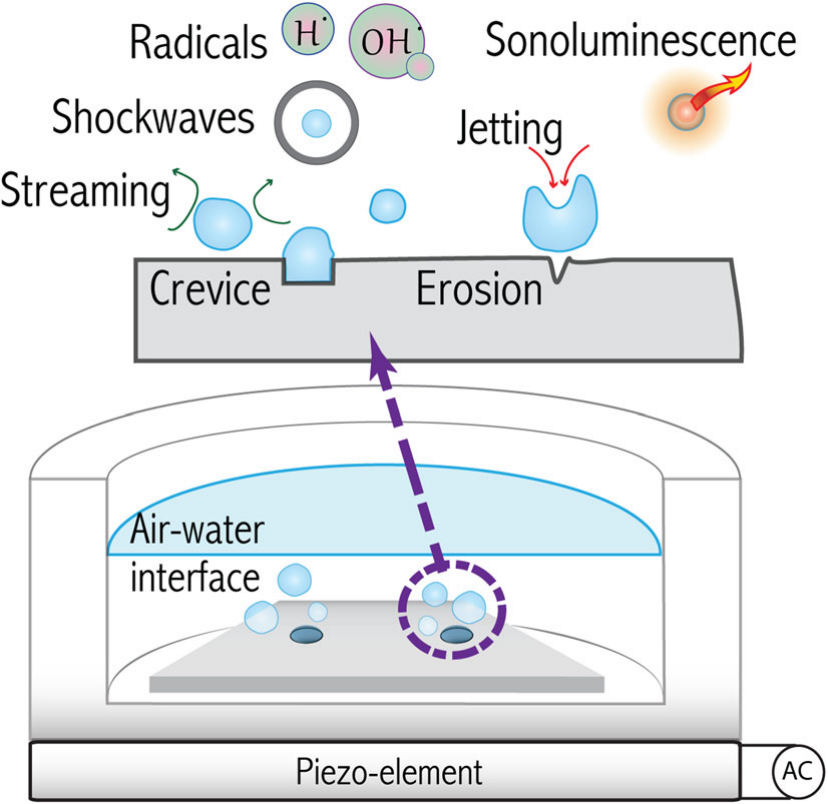
*Top* Diagram illustrating the main phenomena associated to acoustic cavitation: radical production, shockwaves, sonoluminescence, jetting, and erosion. Bubbles are formed from crevices existing in the walls of the reactor or dissolved solid particles. *Bottom* A tailor-made ultrasonic setup in which a transducer (piezo-element) converts electricity into mechanical oscillations that are transferred to the liquid contained in the reactor. This configuration has been used for microfluidics studies

In designing the experimental setups that researchers have used to perform microfluidics and sonochemistry we can identify two main directions based on the final objective of the researchers and availability of resources of each group:

- Use of commercial existing equipment, where a capillary or microfluidic device is placed inside ultrasonic baths or in close contact with ultrasonic horns (see Fig. 1) [82–84].
- Tailor made ultrasonic setups, where a transducer or set of them are glued or clamped against the microfluidic device (see Fig. 4 Bottom) [85–90].

While using commercial devices will lead to fast implementation in the lab, bespoke setups allow an additional degree of customization and optimization in terms of energy efficiency, but also reaction yield. One key design parameter is the coupling between the ultrasound transducer and the microfluidic reactor [91], and such designed systems can then be applied to microfluidic liquid–liquid extraction [89], degradation of contaminants [92–94], and particle synthesis [95, 96].

The large number of techniques at hand and disparate existing knowledge on its effects, has detrimentally influenced the modest utilisation and adoption of ultrasonic cavitation and microfluidics in industry. Furthermore, the differences in experiments designed by chemists and physicists are as varied as the ways a lab researcher and a plant engineer interpret their results. Because of the complex interrelation of physicochemical phenomena resulting from the collapse of a bubble or a cluster of bubbles, replicating results and the choice of quantification techniques (calorimetry, chemical dosimetry, acoustics, optics, etc.) has been troublesome [97–103]. In the particular case of sonochemistry, it has reached the point of being labeled a “black art” [104]. Overall, there is an increasing interest in exploring the potential positive results brought up by combining these two techniques (ultrasound and microfluidics), as observed in specialized conferences and other media. It is still in its early phases, and the reason why the progress is modest will be made more clear in the subsequent sections.

### Entangled Effects of Cavitation

Acoustic cavitation can produce phenomena difficult to explain since it has interconnected variables with non-linear dependencies. The acoustic frequency, pressure amplitude, and other complex physicochemical parameters dictate the creation (or nucleation) and dynamic interaction of collapsing bubbles. The collapse of a bubble can be stable or transient, bubbles inside a cluster can be shielded by those outside; interaction forces between the acoustic field and bubbles (Bjerkness forces) are affected by the liquid properties (gas content, surface tension) and the geometry and materials of the reactors, to name a few [105, 106]. All of the above is further complicated “going down” in scale by confinement effects due to small scales, heat, and mass transfer phenomena. For example, when comparing 1D, 2D and 3D equivalent sonochemical reactors [107], an apparent increase in the reaction rate over the volume change was suggested to be due to the relaxation of space confinement when changing from 1D to 2D geometry. The reaction rate increased by 10 times while the volume increased by 57 times from 2D to 3D. A logical explanation was given by the fact that the total volume of a 3D reactor is not used as efficiently as the thinner layered channel, where nodes and antinode planes are not present. The power input dependencies also exhibit behaviours difficult to explain without a proper understanding of the underlying physicochemical mechanisms of cavitation (e.g. adiabatic compression of gas content dependence on the maximum and minimum radius, number, and spacial distribution of bubbles, shielding effects, etc.). In the same study, a decrease in the production rate of hydro-terephthalic acid (HTA) at higher input power density was found for 2D and 3D, but not in the 1D channel. In later studies, other puzzling correlations of power and an unexpected drop in radical production efficiency [85, 108] were explained by the change in the sphericity of bubble collapses. More details will be provided in the following Sect. 3.3.

From the chemical engineering and practical point of view, the most difficult hurdle for the wide acceptance of ultrasound and sonochemistry as a useful tool has been the measly energy efficiency values. The acoustic transducers transform electrical power into mechanical energy which is transmitted to the liquid. Part of the energy generates cavitation and another heats the whole system, hence not all of the energy produces the desired chemical and physical effects, making it difficult to establish a robust energy balance. For simplification purposes and using a relation reported in the literature [82, 109], we can define the sonochemical efficiency (or yield) as $X_\mathrm{US}=$ measured effect/input power; which in our particular case we define it as:

3 $$\begin{aligned} X_\mathrm{US}=\frac{\Delta H (\Delta N_\mathrm{rad}/\Delta t)}{P_\mathrm{US}} \end{aligned}$$

where $\Delta H$ is the energy required for the formation of OH· radicals, which is equal to the enthalpy of formation of the chemical reaction with a value of 5.1 eV per molecule [110]:

4 $$\begin{aligned} H_2O \mathop {\rightleftharpoons }\limits ^{\Delta H= 5.1\,\mathrm{eV}}OH\cdot + H\cdot \end{aligned}$$

$P_\mathrm{US}$ is the electric power absorbed by the transducer which can be obtained from the measured voltage, current and their phase difference. This is clearly an underestimated value that can help in practical as well as in academic comparisons. Other “measured effects” can be used depending on the specific case, e.g. color dye degradation, mixing efficiency, calorimetric measurements. Depending on the specific study, on average the values reported for OH· radicals are in the order of $X_\mathrm{US}\sim \mathcal {O}(10^{-6})$ [4, 85, 100, 111].

Ultrasonic cavitation is known to be difficult to reproduce since bubbles are normally created from impurities randomly distributed inside the reactor. Impurities such as defects on the walls (crevices) or dissolved solid particles are efficient traps for gas nuclei, and the acoustic nucleation threshold for bubbles trapped in cavities has theoretically and empirically been predicted  [29, 112, 113]. The event of nucleation of a bubble from a crevice serves as a seed for subsequent cavitation. Depending on the acoustic conditions characteristic clouds of bubbles, also known as streamers, can persist for long periods [106, 114–117]. Effects such as inertia, surface tension, and viscous forces in a liquid influence the generation of micro-bubbles and have been studied with the help of non-dimensional numbers such as the ultrasound Weber number (We) and the ultrasound Womersley number (Wo).

5 $$\begin{aligned} \mathrm{We}=\rho f^{2}d_\mathrm{in}^{3}/\sigma \end{aligned}$$

6 $$\begin{aligned}  \mathrm{Wo}=d_\mathrm{in}(f/\nu )^{1/2} \end{aligned}$$

where $\rho$ is the density of the liquid, *f* is the ultrasound frequency, $d_\mathrm{in}$ is the diameter of the pinned bubble, $\sigma$ stands for the interfacial tension and $\nu$ is the kinematic viscosity. The ratio of inertial and surface tension forces is given by the Weber number, We, and the Womersley number, Wo, represents the ratio of pulsatile to viscous forces. According to a particular study [114], a uniform diameter of bubbles is obtained when $8.16< \mathrm {We} < 300$ and $2< \mathrm{Wo} < 5$. For $\mathrm{Wo} > 5$, the inertial effect dominate the viscous effect, resulting in bubbles of various sizes being released from the gas-liquid interface. When $\mathrm{Wo} < \,2$, the interface was not distorted sufficiently to release bubbles. When $\mathrm{We} > 300$, the inertia dominates surface tension effects producing bubbles of various sizes. For small We, the interface oscillations are stable without strong distortion, and no bubbles are produced due to dominating surface tension effects.

In general, it has been possible to study to a good level of detail physicochemical exotic phenomena such as plasma formation inside the bubbles, the emission of light (sonoluminescence), radical production, shockwaves, streaming and jetting (see Fig. 4) [11, 98, 106, 111, 118–120]. The interaction of individual bubbles or clusters as they collapse among themselves and against nearby surfaces (see Fig. 2) has also been studied given its importance for applications such as erosion prevention, cleaning, surface modifications, biology, and several chemical processes [13, 121–126].

**Fig. 5:**
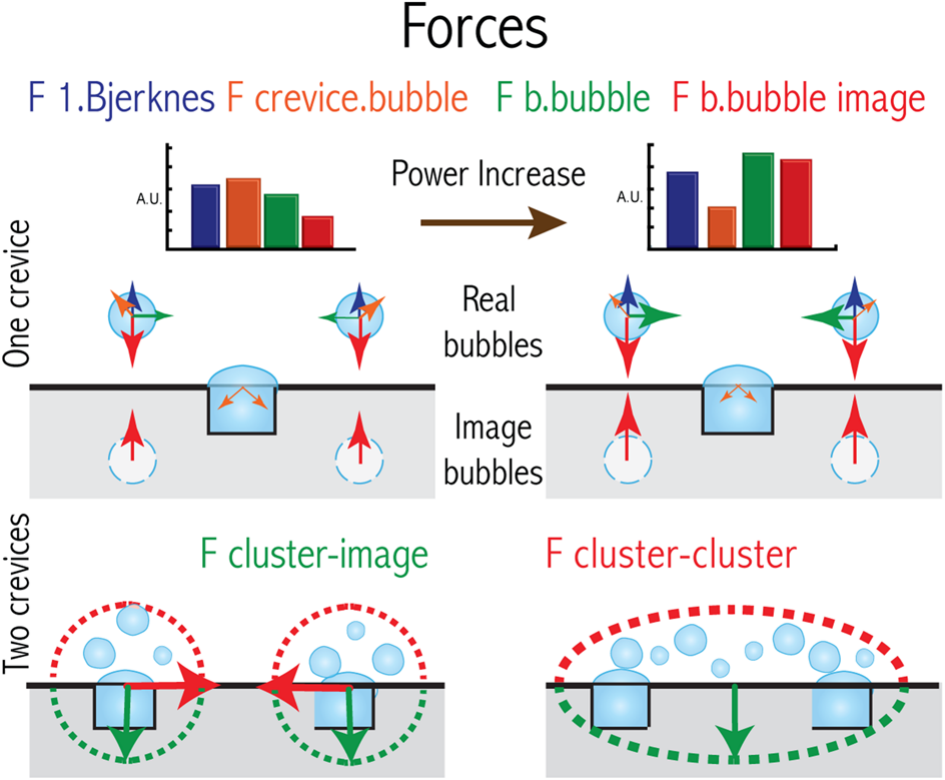
Simplified representation of forces and colors associated to them represented in arbitrary units in a *bar plot* for two different power settings (higher to the *right*). The *top chart* depicts the case of a single crevice and how the bubbles around it interact with each other. The *bottom chart* shows similar information, but when two crevices are etched and how the clusters of bubbles interact (see Fig. 6 for more details)

In the particular cases that acoustic cavitation takes place close to a surface (see Fig. 5), interaction forces among the bubbles and the acoustic field alter the otherwise “ideal” spherical collapse. Oscillating bubbles, and bubble clusters oscillating close to a surface are attracted to their “image” on the virtual mirrored space. Jets and shockwaves emitted at different instants during acoustic cavitation are the main mechanisms responsible for the erosion of surfaces (see Fig. 4)  [6, 13, 122, 127].

### Controlling Cavitation with Microscopic Crevices

For a newcomer to the sonochemistry realm, it is important to understand that the rationale behind all the effects of cavitation begins with considering each bubble as a reactor in itself. Then, a working ultrasonic bath or horn will produce an undetermined number of bubbles of different sizes, and consequently each collapse will result in different temperatures and pressures inside of the bubble. It should be no surprise that as a result, the chemical or physical effects have a broad distribution of values, simply because all bubble-reactors behave different.

Despite the careful experimental precautions, the number of bubbles can be underestimated since some large bubbles overlap, and many smaller bubbles are not counted due to the optical resolution [108]. Nevertheless the qualitative and quantitative measurements of their number, coupled to the radical formation values can give relevant information to compare with other reports. Dividing the radical production per cycle by the average number of bubbles per cycle, gave bubble radical productions in the same order of magnitude measured by Didenko and Suslick [111] who reported data for a single bubble of maximum radius of 28.9 $\upmu$m driven at 52 kHz producing OH· radical generation at a rate of 6.6 $\times$ 10$^5$ per cycle. These values for radical production can be taken as a reference of the order of magnitude that can be produced by a generic bubble in an ultrasonic reactor [128].

The quest to trap bubbles for the study of nucleation and collapse was initially driven by fundamental research questions. Different approaches have been used, from acoustic trapping to geometrical confinement of conical bubbles or micromachined crevices [86, 111, 114, 129, 130]. Focusing on aspects relevant for the chemical engineering community, we will elaborate on a series of recent results motivated by two practical questions:

1. How can the energy efficiency of sonochemical reactors be improved?
2. How can bubble generation be controlled reproducibly?

The challenge was, not surprisingly, conquered as a result of an interdisciplinary collaboration between microfabrication experts, physicists, and chemists. A microfluidic sonochemical reactor was designed, modeled and tested under laboratory conditions [85, 108, 131, 132], and more recently the same concept has been patented, scaled up, numbered up, and commercialised [133–135]. We consider this case a good example of the positive outcome of the synergy of microfluidics, ultrasound and process intensification concepts.

#### Batch Micro-Sono-Reactor

The structure of a batch ultrasonic reactor was modified by micromachining artificial crevices of ca. 30 $\upmu m$ diameter and 10 $\upmu m$ depth, unto the surface of silicon substrates. This passive modification was used as a trap for a determined number of bubbles (see Fig. 4). Upon sonication, the bubbles served as a seeding gas volume that ensured continuous formation of smaller bubbles (streamers) containing a mixture of gas and water vapor. This continued formation of bubbles can be sustained by virtue of a phenomenon termed “rectified diffusion” [27, 136]. This phenomenon occurs as a bubble expands, and gas-solvent molecules diffuse–evaporate into the bubble; conversely, when the compression part of the sound field arrives, gas-solvent molecules diffuse–condense out of the bubble. Because of an unbalance in the area in the expansion (larger) and compression phase (smaller), there is a net gain inside the bubble which can compensate any mass loss as the smaller bubbles are created from the crevice.

**Fig. 6:**
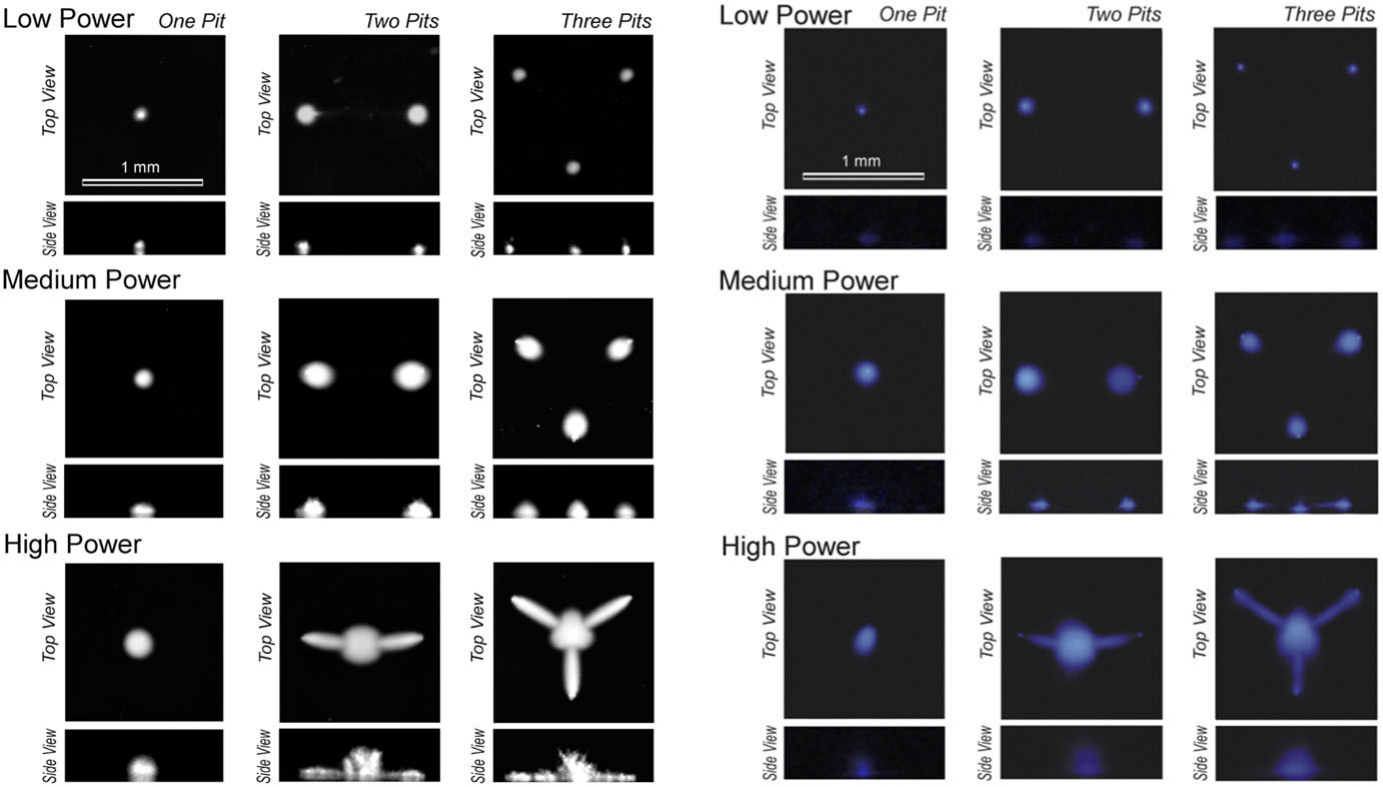
*Left* Images showing* top* and* side* views of the bubble structures generated from micromachined silicon surfaces for different configurations (1, 2, and 3 crevices) and for increasing power level. Low (electrical) power corresponds to 74 mW, medium power to 182 mW, and high power to 629 mW. *Right* Corresponding Luminol luminescence in dark room conditions. The *scale bar* represents 1 mm of a microreactor with total volume of ca. 250 $\upmu \ell$. Reproduced with permission from [85]. Copyright 2010 Wiley-VCH Verlag GmbH & Co. KGaA

In the first report of a series of studies based on the same device [85], the streamers were demonstrated to produce hydroxyl radicals (OH·) by imaging its reaction with luminol. The reaction rates were measured as a function of electrical power with terephthalic dosimetry (see Fig. 6).

**Fig. 7:**
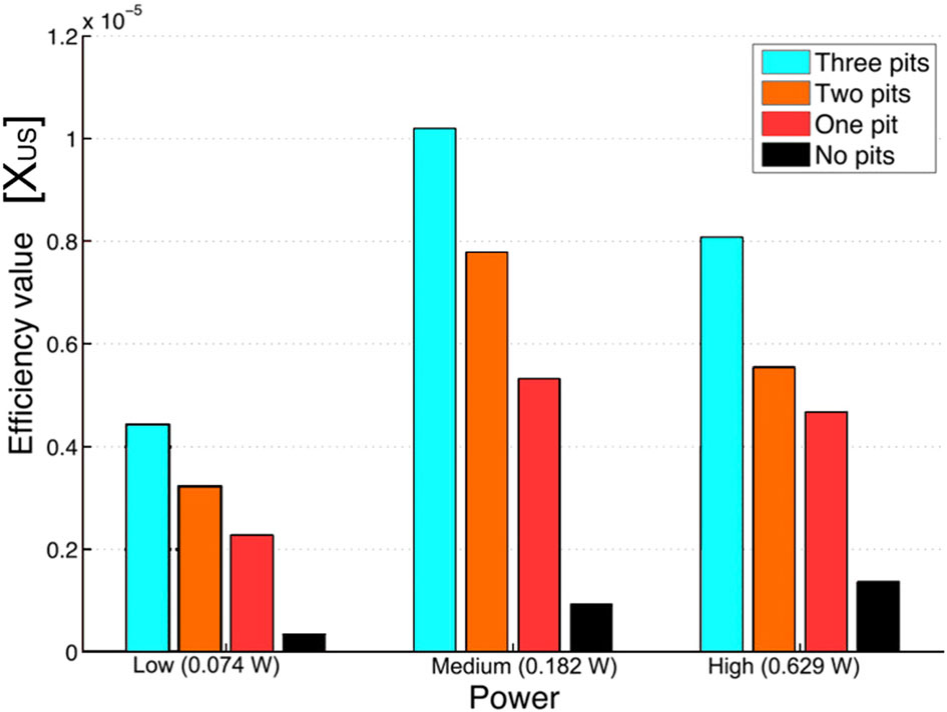
Experimental efficiency values ($X_\mathrm{US}$) for different number of crevices and different US powers calculated from Eq. (3) [108]. For each power the presence of crevices results in an increase in efficiency as the number of crevices is increased. As the power is increased from low to medium the trends increase for any number of crevices, except for high power, as it seems to decrease in all cases. Whether this result corresponds to true differences in the acoustic and sonochemical processes or to a decreased efficiency of conversion from electrical to mechanical power has not been clarified. Reprinted from [108]. Copyright 2013, with permission from Elsevier

As introduced in Sect. 3.2, though the efficiency when crevices were present was higher at the three powers tested (about one order of magnitude), at the highest power there was an unexpected drop. The efficiency values calculated with Eq. (4), are shown in Fig. 7. From a speculative analysis it was concluded that at high power, when the bubble pattern changes (see Fig. 6) a different radical generation distribution over the reactor volume could take place. Looking at each bubble as individual reactors, the energy efficiency of each collapse is strongly determined by the sphericity of the collapse, and the maximum size reached before collapsing. The smaller bubbles were thought to be stiffer due to a surface tension contribution and were not expected to grow considerably large during expansion. This leads to a consequently weaker compression and lower maximum temperatures reached after each collapse. Conversely, larger bubbles do not collapse spherically especially when close to a solid surface, hence limiting its maximum compression potential.

**Fig. 8:**
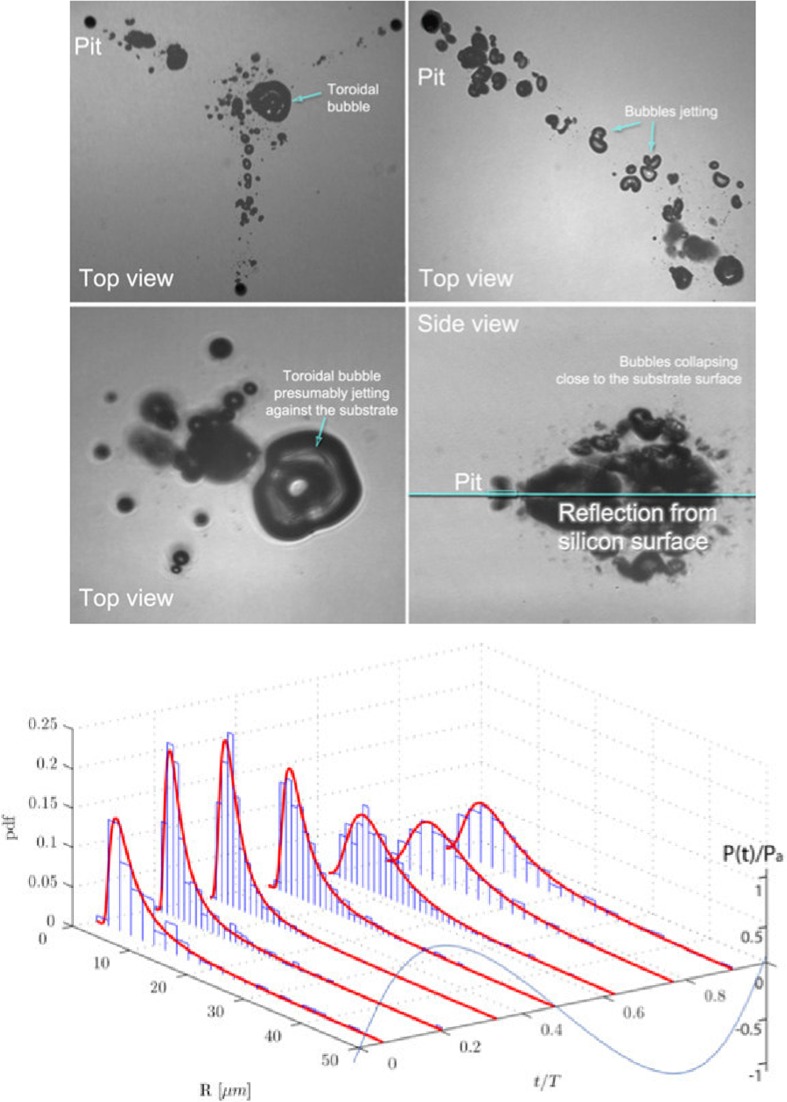
*Top* Short time exposure image at high power settings (0.981 W) where the deformation of bubbles and jetting phenomena is visible. *Bottom* Bubble size distribution histograms at a power of 0.981 W for three pits. The axis to the *extreme right* represents the normalized pressure for the acoustic cycle. Reprinted from [108]. Copyright 2013, with permission from Elsevier

To clarify these assumptions, two complementary studies were devised. In the first one, the light emission from the collapse of bubbles (sonoluminescence) and as the result of the reaction of OH· with luminol (sonochemiluminescence) was measured with a photomultiplier [131]. Transient cavitation conditions were verified by measuring the SL intensity in propanol solutions. The different light intensities of SL and SCL helped to establish a difference in the bubble population able to emit light and those chemically active. This type of photosensitive study is important when is impossible to capture the fast dynamics of bubble collapse with the available equipment. The second study was directed at taking short exposure images and fast-imaging movies for the determination of the number and sizes of bubbles [108]. The quantification of radicals was correlated with the number of bubbles and radius-time evolution in an acoustic cycle. The main conclusions from this study were that at higher powers, hence larger pressure fields in which bubbles expand and collapse, the shape of each bubble deviates from the ideal sphericity which corresponds to a maximum conversion of potential energy into sonochemical effects (shockwaves, liquid jets, sonoluminescence, and radical formation, see Fig. 8). Those bubbles that collapse towards each other, or in the proximity of an interface, deform and result in jet formation. As a result, not all the energy that could heat the bubble content is available for the rupture of chemical bonds, such as that of water molecule which is measured by the formation of OH· radicals.

From the reactor design perspective, the fact that bubbles remain close to the surface from which they were created can have negative consequences. With this microreactor that allows control over cavitation, the erosion caused by bubbles collapsing in the vicinity of different silicon substrates could be studied right from the initial incubation period and through more advanced stages [127]. The same effect of various sources of damage formation such as jetting, shock waves, direct bubble impact, and surface stress corrosion that can cause the damage observed for three crystallographic silicon surfaces studied, was later used for another useful application: the rapid removal from substrates of deposited organic and inorganic materials, such as biofilms [137]. The practical relevance of these results will be continued in the following subsection.

Contrary to what happens with most commercial ultrasonic equipment, tailor-made devices can in principle be operated at different frequencies. The studies of this micro-sono-reactor were conducted at one frequency (200 kHz) where it was more active. Based on what was discussed in Sect. 3.2, for the case of water and air on a 30 $\upmu \mathrm{m}$ crevice we have $\mathrm{We}=15$ and $\mathrm{Wo}=13.4$, indicating that relatively small interface oscillations of the bubble in the crevice (small We) are unbalanced by inertia dominated effect resulting in bubbles of different sizes (large Wo). Snapshots of videos from experiments of this system indeed show significant bubble deformations of the bubbles ejected from the bubble stabilised on the crevice (see Fig. 8) [108, 117]. We have not found in the literature other studies reporting on such large values for both numbers, hence a parametric study in this direction would be highly valued for future applications. Similar studies addressing the effect of different frequencies in the same reactor will shine more light into the challenging “black” field of sonochemistry.

#### Scaled-Up Non-Conventional Batch Reactor

Scaling-up or numbering-up are the most frequent strategies used in microfluidics whenever there is an interest in practical applications beyond the lab-scale. Small volumes ca. 250 $\upmu \ell$, such as the ones used in the studies mentioned in the previous sections are of limited relevance for practical or industrial uses. Making larger crevices or higher amplitudes to obtain larger bubbles before collapse does not correlate linearly with better results, as explained in Sect. 3.3.1. Additionally, despite the unique control over the location of bubble clouds, it is difficult to envisage any commercial appeal that the presence of a few crevices etched on the bottom of a batch reactor can produce. Even when the numbering-up of crevices might be possible by drilling crevices on the surfaces of ultrasonic baths or the tips of horns, it seems like an improbable option for all manufacturers of baths and horns.

**Fig. 9:**
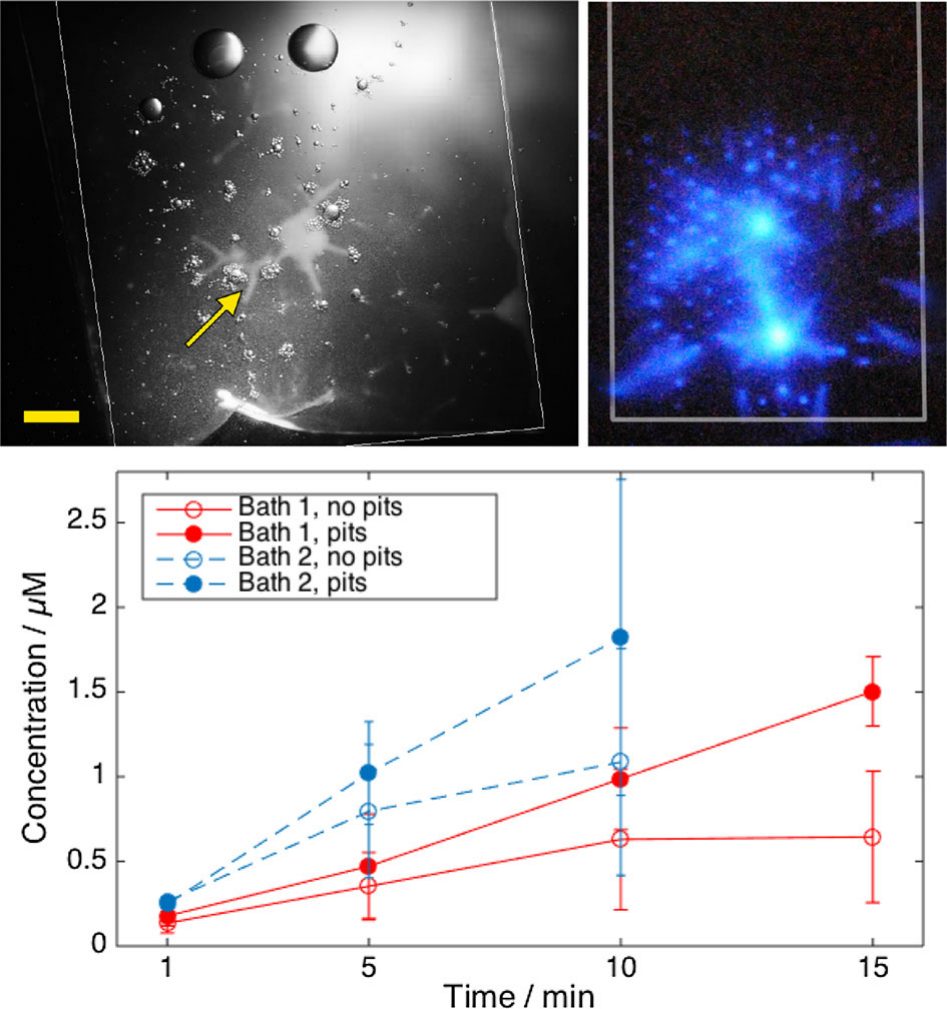
*Left* Clouds of bubbles inside a cavitation intensifying bag; *right* sonochemiluminescence as a result of the radicals produced by the bubbles upon reaction with luminol. The *arrow* indicates the bubble clouds emerging from the crevices. The *scale bar* corresponds to 5 mm. *Bottom* Radical production in bags with and without pits, as a function of time and for a large (45 kHz, 364 kPa and 33.3 W/L) and a small (35 kHz, 427 kPa and 24.2 W/L) ultrasonic bath. Reproduced with permission from [134]. Copyright 2010 Wiley-VCH Verlag GmbH & Co. KGaA

**Fig. 10:**
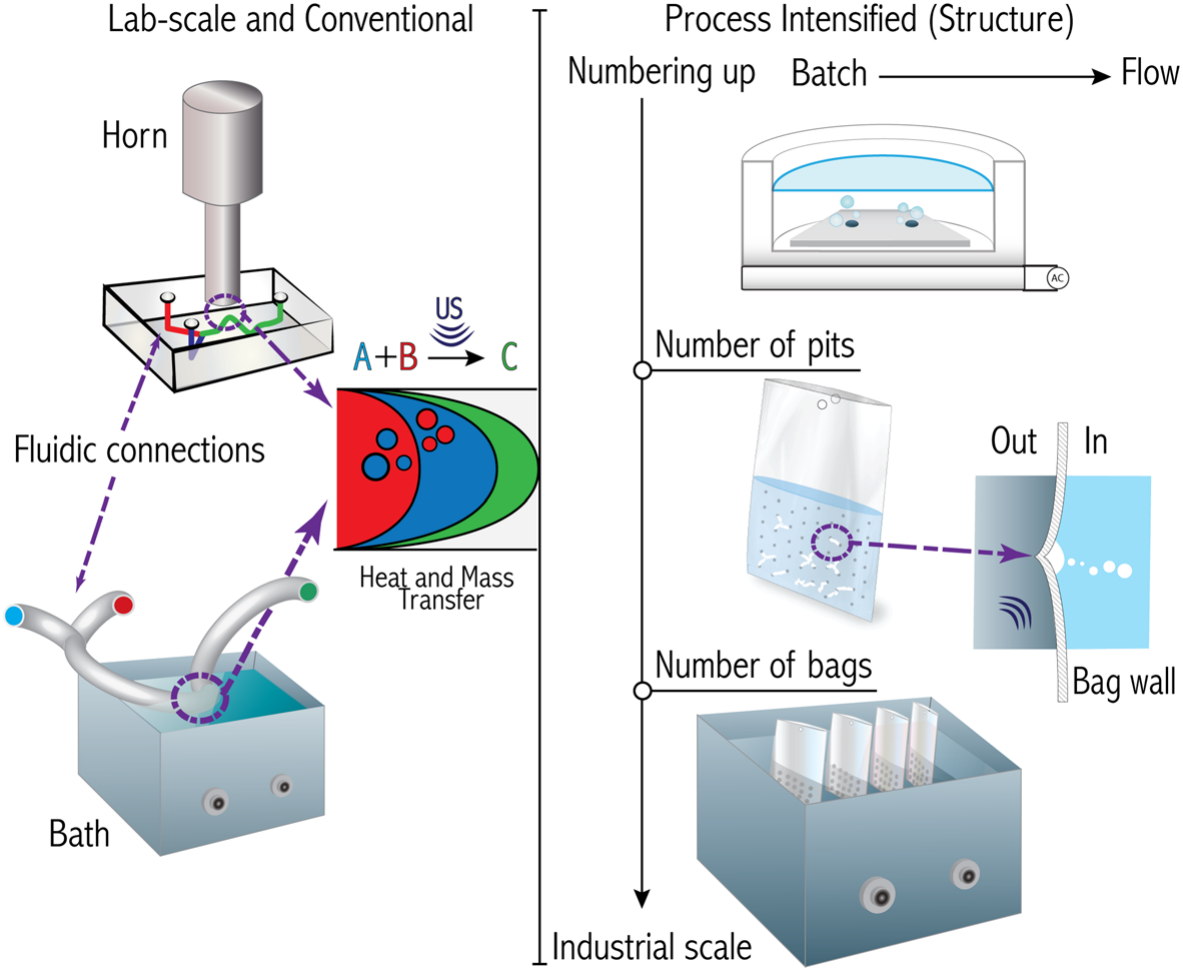
*Left pane* conventional and lab-scale uses in which existing ultrasonic devices such as horns and baths are used to promote reactions. *Right pane* An example of scaling-up a micro-sonochemical reactor by increasing its volume and numbering-up the amount of crevices and the Bubble bag

In trying to find a universal solution that could be used in most ultrasonic equipment, the new concept of the Bubble Bag was devised [134]. The inner surface of a bag that serves as a container can be indented with crevices for gas entrapment (see Fig. 9). It has been described as a cavitation intensifying bag since it enables a production of bubbles that does not takes place on a simple bag. The Bubble Bag can be interpreted as sophisticated beaker, initially made of plastic, but it can be manufactured in any other material since the principle for creating bubbles from crevices has already been demonstrated in silicon and glass. The first and most versatile advantage is that it does not depend on a particular frequency for cavitation to happen as demonstrated with several pieces of ultrasonic equipment; see in Fig. 9 the results of two ultrasonic baths with different frequencies with and without crevices. The second advantage schematically represented in Fig. 10 is that numbering-up of such a beaker is straightforward.

Designed originally for cleaning arbitrary objects [135], the current dimensions and potential use for radical generation allows us to classify it as a milli-reactor [138]. Using the same configuration, the bags can also be used for other applications such as emulsification, and presumably for any physicochemical processes where ultrasound is currently used [139, 140]. The most relevant scientific advantage is that reproducibility of results and energy efficiency are considerably better than when compared with a bag not having crevices. The standard deviation of radicals detected was reduced by 22 %, accompanied by an increase of 45.1 % in efficiency (see Fig. 9). The number of pits and bags can be in principle as large as the ultrasonic equipment allows for. New optimisation and characterisation studies will be required to find the best position or configuration of transducer-bag positioning. So far, the Bubble Bag has been operated in batch mode; we expect to provide more results as a flowing reactor in the near future in a multiple-stage assembly. We believe this concept will lead to new applications and improved versions to be reported in the specialised literature.

To conclude with this section, in Fig. 10 we illustrate the trends identified up to now and where we believe the future developments will head to: intensified continuous flow and industrial scale sonochemical reactors.

### Acoustic Streaming

A phenomena increasingly exploited in microstructured devices is acoustic streaming. In general terms, acoustic streaming is the generation of a convective motion (i.e. fluid flow) through the presence of an acoustic field [141]. Real fluids exhibit a viscous attenuation towards an acoustic wave traveling through them, which in the case of an oscillatory acoustic field results in a time-averaged displacement of individual fluid elements. This local displacement can develop into a steady fluid flow, termed acoustic streaming [142]. Depending on the location and length-scale of the fluid motion several sub-categories of acoustic streaming can be defined, such as boundary layer driven streaming (Schlichting and Rayleigh streaming), Eckart streaming in the bulk fluid, and cavitation microstreaming [141, 143]. In the following we will discuss each streaming phenomena and provide application examples in microfluidics.

*Boundary layer driven streaming* Because of the no-slip condition at a solid interface, the fluid flow in the boundary layer is characterized by a steep velocity gradient. In turn, this steep velocity gradient is also responsible for a larger viscous dissipation of acoustic energy compared to bulk flow [144]. Applying a standing acoustic wave parallel to the solid interface results in spatially fixed pressure nodes and antinodes, which leads to a steady vortical fluid motion in the boundary layer (termed Schlichting streaming). These vortices in the inner boundary layer will excite counter-rotating vortices in the outer boundary layer, and this induced streaming motion is termed Rayleigh streaming [145].

Boundary layer driven streaming in microfluidics is often employed for particle trapping, e.g. to either control local particle concentration [146], particle aggregation [147], or to separate particles based on their size (in combination with the acoustic radiation force acting on the particles) [54, 143, 148]. However, it can also be used to overcome one of the disadvantages of microstructured devices, i.e. that the only means of mixing is by diffusion due to the predominant laminar flow. Rayleigh streaming has been shown to improve micromixing [149].

*Eckart streaming* When the dissipation of the acoustic energy takes place in the bulk of the fluid, Eckart streaming is observed. However, as microstructured devices are characterized by a large surface-to-volume ratio, their hydrodynamics is governed by boundary layers, and bulk flow is rarely encountered [141]. In general, Eckart streaming will only occur when high frequency ultrasound is applied, and when the characteristic dimension (defined as the propagation direction of the acoustic wave) is in the order of millimeters.

Consequently, the applications of Eckart streaming are limited in microfluidics. However, based on the experimental observation that the resulting convective motion is characterized by high velocity jets [150], it has been used to design a valveless ultrasonic pump [151].

*Cavitation microstreaming* Cavitation microstreaming is generated by the acoustically driven oscillations of microbubbles in a liquid. These oscillations are transferred via the boundary layer surrounding the bubble and generate vorticity and convective motion in the fluid [152]. In addition to the streaming motion, oscillating bubbles will also create stress fields in their surrounding, which largely depend on their mode of oscillation [153].

These stress fields coupled with the streaming effect enable the therapeutic use of microbubble-mediated ultrasound. An increased permeability of cell membranes has been observed, which allows e.g. targeted drug delivery. In addition, the combination of microbubbles and ultrasound have been shown to accelerate the breakdown of blood clots (thrombolysis) [154]. Furthermore, cavitation microstreaming is also applied to enhance mixing in microfluidic devices [155].

### Clogging Prevention

In this section we will review the handling of solids in microfluidic systems. The solid material can either be comprised of an unwanted and insoluble by-product of a reaction, or the target compound (e.g. nanoparticle synthesis or crystallization of organic molecules). In general, managing solid particles in flow represents a major challenge for the upstream, continuous processing of fine chemicals in microreactors [56]. Many synthetic organic reactions either involve the use or the generation of insoluble compounds [57]. In the following we will discuss the ultrasound application strategies to control the particle formation within the microchannel.

The impact of channel clogging on continuous manufacturing is best illustrated with a study conducted at Lonza [156]: In a screen of 86 different reactions it was found that 59 % would benefit from a continuous process, however, this number reduced to 19 % due to the presence of solids. Consequently, to assist the transition from conventional batch to continuous manufacturing processes exploiting microreaction technology, reliable solids handling needs to be established.

**Fig. 11:**
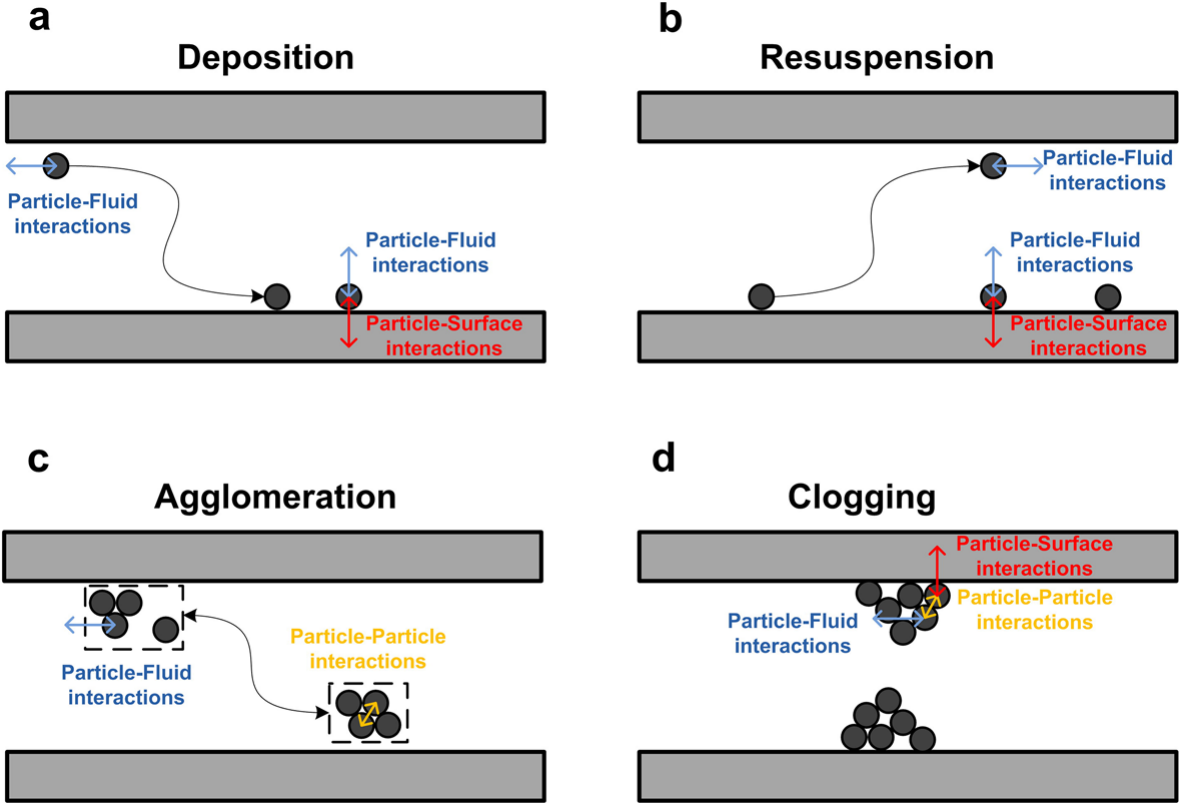
Interactions governing the behavior of solid particles in microchannels [58]. Particle-fluid, particle-particle, and particle-surface interactions are always present, but their relative importance differs widely depending on the considered case (deposition, resuspension, agglomeration, and clogging). Reproduced with permission from [58]. Copyright 2014 Tekno Scienze Srl

Figure 11 depicts the main interactions governing particle behavior in microfluidic channels, namely particle-fluid, particle-particle, and particle-surface interactions [58].

The relative importance of these interactions differs on a case by case basis, which leads to several phenomena discussed below: Deposition of particles (Fig. 11a) is initiated by particle-fluid interactions transporting the solid to the microchannel wall where it finally sticks due to a dominating particle-surface interaction. Increasing the particle-fluid interaction by e.g. increasing the fluid velocity will lead to resuspension (Fig. 11b). The particles will agglomerate in the bulk of the fluid by particle-particle interactions (Fig. 11c); however, agglomerate break-up can again occur when the particle-fluid interactions overcome the inter-particle interactions. As we will discuss later, this is one of the main avenues where ultrasound comes into play, as the induced cavitation will give rise to hydrodynamic forces on the agglomerates and thus decrease their size. The clogging phenomena itself (Fig. 11d) is governed by all three interactions, and usually occurs via bridging of a constricted microchannel cross-section [56, 57].

The most used passive means to prevent microchannel clogging are:

- Application of two-phase flow, allowing the introduction of a secondary phase which dissolves the solids. However, this also introduces additional mass transport limitations.
- Increasing the wall shear stress by increasing the fluid velocity.
- Reactor surface modification, mostly based on fluoropolymers, to achieve non-sticking reactor walls.

The passive techniques are reviewed elsewhere [56–58], here we primarily focus on active clogging prevention using ultrasound.

The integration of acoustic actuators with microstructures is a new and emerging area, where the acoustic energy is mostly supplied using transducers or piezoelectric microdevices with different sizes and geometries [77, 157–161]. One application of ultrasound integration in microfluidics is the excitation of a standing acoustic wave in the fluidic channel [53]. Particles in an acoustic standing wave field will experience an acoustic radiation force [54], which can be exploited for the manipulation of their trajectories [162]. This concept can even be extended to controlling the trajectory of single bubbles [163].

At increased power, acoustic irradiation has been shown to be successful in reducing agglomerate particle size, which is essential to prevent clogging [158, 159, 164]. A well studied reaction system and also a challenge under flow conditions due to clogging is that of Pd-catalyzed CN cross-couplings [158]. Under typical reaction conditions inorganic by-products precipitate immediately in the non-polar solvents needed for this transformation. Furthermore, the recent developments of highly active palladium catalysts which allow for extremely fast reactions, and thus also a fast generation of these inorganic salts.

**Fig. 12:**
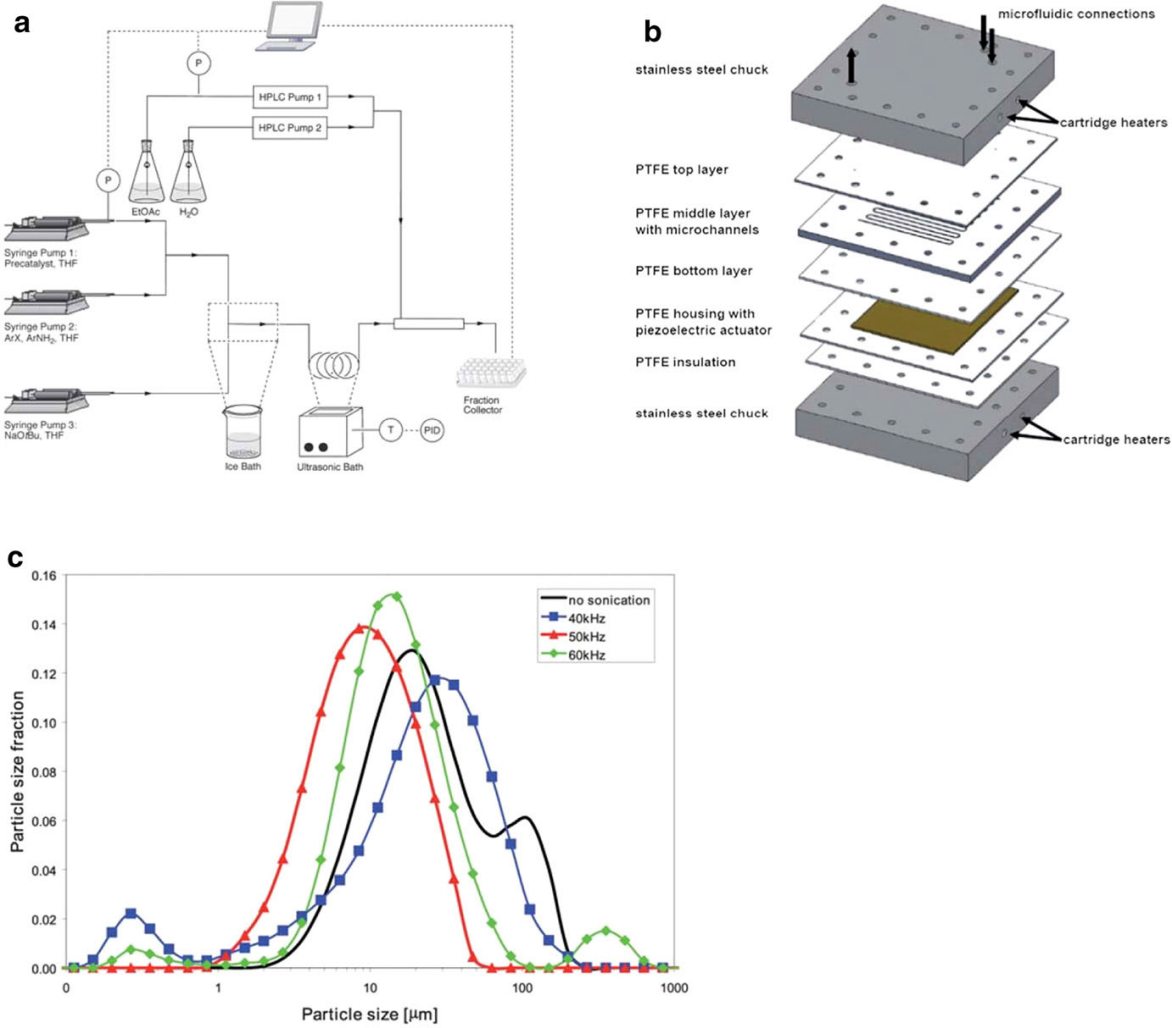
Examples of the use of ultrasound in microfluidic systems to prevent clogging. The methods of ultrasound integration range from immersing the reactor in an ultrasonic bath (**a**) to a full reactor assembly with the ultrasound transducer positioned next to the microfluidic channels (**b**). **a** Using an ultrasonic batch to handle solids formed during palladium-catalyzed amination reactions [159]. Reproduced from [159] with permission of The Royal Society of Chemistry. **b** A Teflon microreactor with integrated piezoelectric actuator to handle solid forming reactions [160]. Reproduced from [160] with permission of The Royal Society of Chemistry. The latter design eliminates the need of a transfer medium for the acoustic wave, and allows precise control of the applied ultrasound frequency and power. **c** The final precipitate particle size depends on the applied ultrasound frequency, and consequently a precise control of the operating parameters is desired for the combination of ultrasound and microfluidics [160]. Reproduced from [160] with permission of The Royal Society of Chemistry

One approach to prevent clogging is to immerse Teflon tubing in an ultrasonic bath for irradiation, as shown in Figs. 10 and 12a [159] and discussed in Sect. 3.1.

**Fig. 13:**
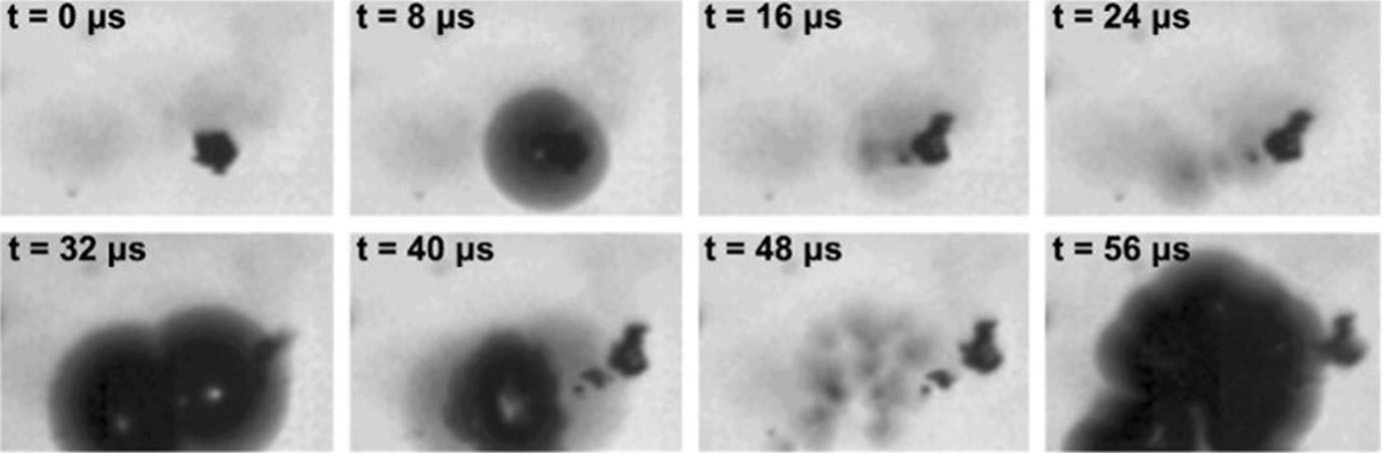
Images of an oscillating cavitation bubble that nucleated on the surface of a calcite crystal. The single crystal fragmentizes as a result of the violent collapse of the bubble [165]. Reprinted from [165]. Copyright 2013, with permission from Elsevier

However, when using an ultrasonic bath, one has to be aware of the fact that not a single frequency is excited, but the resulting waveform can be quite complex [158]. Furthermore, the emitted ultrasonic waves first need to couple with the media in the bath before transferring to the microreactor. As such, integrating a piezoelectric actuator directly into the microfluidic assembly to directly transmit the acoustic waveform to the reactor is energetically more efficient, as mentioned in Sect. 3.1. An example of such a layered microreactor system is shown in Fig. 12b, which was also successfully applied to the aforementioned Pd-catalyzed CN cross-couplings and allowed for long term operation [160]. High-speed imaging revealed the formation of gas bubbles upon ultrasonic irradiation via the mechanism of stable cavitation, and the pressure forces associated with this formation lead to the breakup of the particle agglomerates [165–167]. This phenomena is clearly depicted in Fig. 13, which shows the fragmentation of a calcite crystal due to the collapse of a bubble.

Furthermore, using piezoelectric actuators allows for a precise control of the operating frequency, which is important to control the resulting size of the agglomerates. Figure 12c depicts the particle size distribution of inorganic precipitates subject to the applied ultrasound frequency, and for the particular setup the identified optimum frequency corresponded to 50 kHz.

### Microfluidics As a Tool for Particle Synthesis

Microfluidic systems have also seen an increase in use for cases where the particulate matter is the desired product. Continuous manufacturing enables the formation of particles with narrow size distribution, which is an important property for their final application. In general, particles of similar properties will be formed when they experience similar conditions in the reactor, which is difficult to achieve in batch systems due to mixing limitations associated with their large size, but feasible on the micro-scale using continuous flow.

However, focusing on particle formation processes, these devices need not only overcome clogging of the microchannels, but also ensure kinetic control of nucleation, growth, and agglomeration. The same Teflon reactor design with integrated piezoelectric actuator as outlined above was applied to the crystallization of hydroxyapatite (HAp) in continuous flow [168, 169]. Compared to the batch process (stirred tank reactor), the HAp particles formed were more crystalline and less carbonate contaminated. The sonication strategy also lead to a reduction in particle aggregation and primary particle size; however, the resulting particle size distribution was poly-modal, which strongly suggested that the nucleation, growth and agglomeration processes were not precisely enough controlled in this system. A decoupling of nucleation and growth of particles in the reactor will increase this kinetic control, and one example to achieve this is the use of an ultrasonic horn next to the microchannel [95]. This will create a spatially localized zone within the reactor for the generation of crystal nuclei. Using a similar setup, Rossi et al. [96] studied the effect of supersaturation and ultrasound power on nucleation in more detail, and it was concluded that the transient cavitation of bubbles is a significant mechanism for enhancing crystal nucleation.

## Outlook and Future Applications

We aimed to cover most of the recent examples of the use of microfluidics and ultrasound in which given processes have been intensified. As mentioned in the text, we shared our conclusion that synergy, as described in process intensification terms, plays the largest role in the combination of microfluidics and ultrasound applications.

We have highlighted the emerging availability of commercial devices, but also stated that customisation leads to increased efficiency and product yield. As such, we see the future need for a plug-and-play approach, with readily available off-the-shelf components, which allow custom designed experimental setups. We speculate that market and society requirements will be the drivers for the developments we might see along this line, particularly in the food processing, nanomaterials synthesis, cosmetics, medicine, and other relevant applications.

On the scientific front we identified several developments which warrant further investigation to gain a more fundamental understanding. In terms of cavitation, we pose the following research questions:

- Is it possible to control further processes and applications such as the synthesis of nanomaterials, exfoliation of nanoparticles, or the improved properties of emulsions of relevance in food processing, coatings, etc.?
- How much more can a sonochemical reactor based on artificial crevices be scaled-up efficiently with a continuous flow operation? This is of relevance for applications where large volumes need to be processed as in water treatment or food processing.
- Can the surface of bubbles be further used as a “meeting point” for reactive species, so that bubbles can act as catalytic reaction spots with higher control than currently achieved?

In the field of particle synthesis and solid precipitation the following questions should be addressed:

- What is the influence of cavitation intensity on crystal nucleation rates? Using crevices for obtaining controlled transient cavitation could improve crystal nucleation mechanisms, and hence will be worth addressing in future studies.
- Is there a critical cavitation bubble size to enhance nucleation?
- What is the effect of ultrasound irradiation on crystal growth; e.g. can we exploit acoustic streaming to enhance convective mass transfer to achieve increased growth rates?

Our vision of where the field is going is open to debate and we invite colleagues to enrich it with future ideas.
